# Supplementary material for: Adopting orphan receptors: zebrafish Tlr4 homologs mediate responses to group IX/X transition metals
Source: Front Immunol. 2025 Nov 26;16:1679375. doi: 10.3389/fimmu.2025.1679375 (PMC12689318; doi:10.3389/fimmu.2025.1679375)
Supplement: Supplementary Figure 1 — Multiple sequence alignment and percent identity matrix for TLR4 (full protein) between zebrafish, mice, and humans. (A) The comparison between sequences shows the homology and conservation of residues mainly in the latter third of the protein sequence. Similar residues (100%) are highlighted in black, partially similar residues are highlighted in dark grey (80%-100%) and light grey (60%-80%). The colored histogram shows the mean pairwise percent identity for each column of the alignment, where green represents 100%, yellow represents >30%, and red represents <30%. (B) Table shows the percent sequence identity between mammalian TLR4 (mouse and human) is >60%, while the percent identity between mammalian and zebrafish Tlr4 homologs is <40%. Alignments, similarity, and the percent identity matrix were made and calculated using Geneious Prime. [file Supplementaryfile1.docx]

Supplementary Material

**
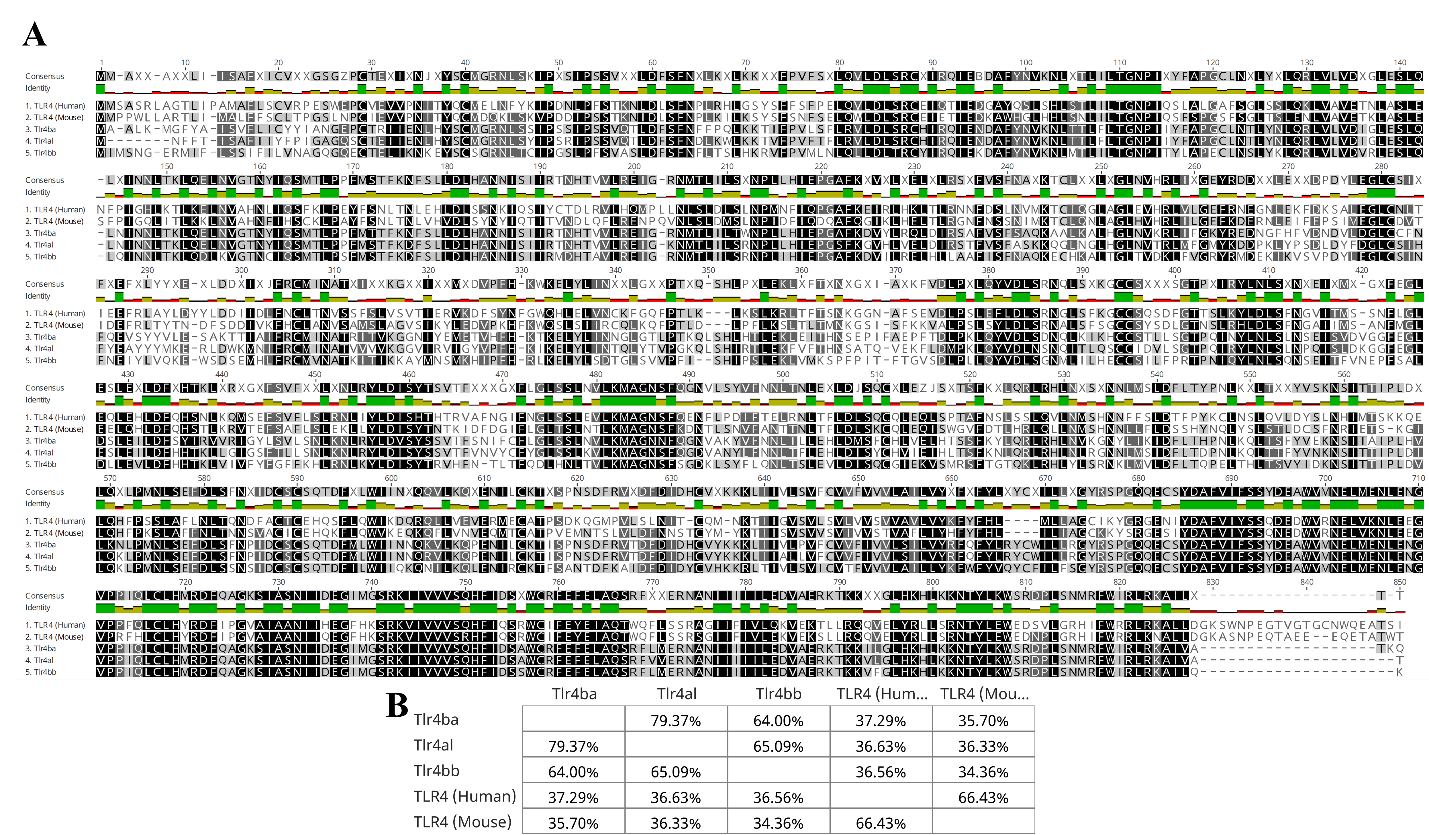
**

**Supplementary Figure 1. Multiple sequence alignment and percent identity matrix for TLR4 (full protein) between zebrafish, mice, and humans.** A) The comparison between sequences shows the homology and conservation of residues mainly in the latter third of the protein sequence. Similar residues (100%) are highlighted in black, partially similar residues are highlighted in dark grey (80%-100%) and light grey (60%-80%). The coloured histogram shows the mean pairwise percent identity for each column of the alignment, where green represents 100%, yellow represents >30%, and red represents <30%. B) Table shows the percent sequence identity between mammalian TLR4 (mouse and human) is >60%, while the percent identity between mammalian and zebrafish Tlr4 homologs is <40%. Alignments, similarity, and the percent identity matrix were made and calculated using Geneious Prime.

**
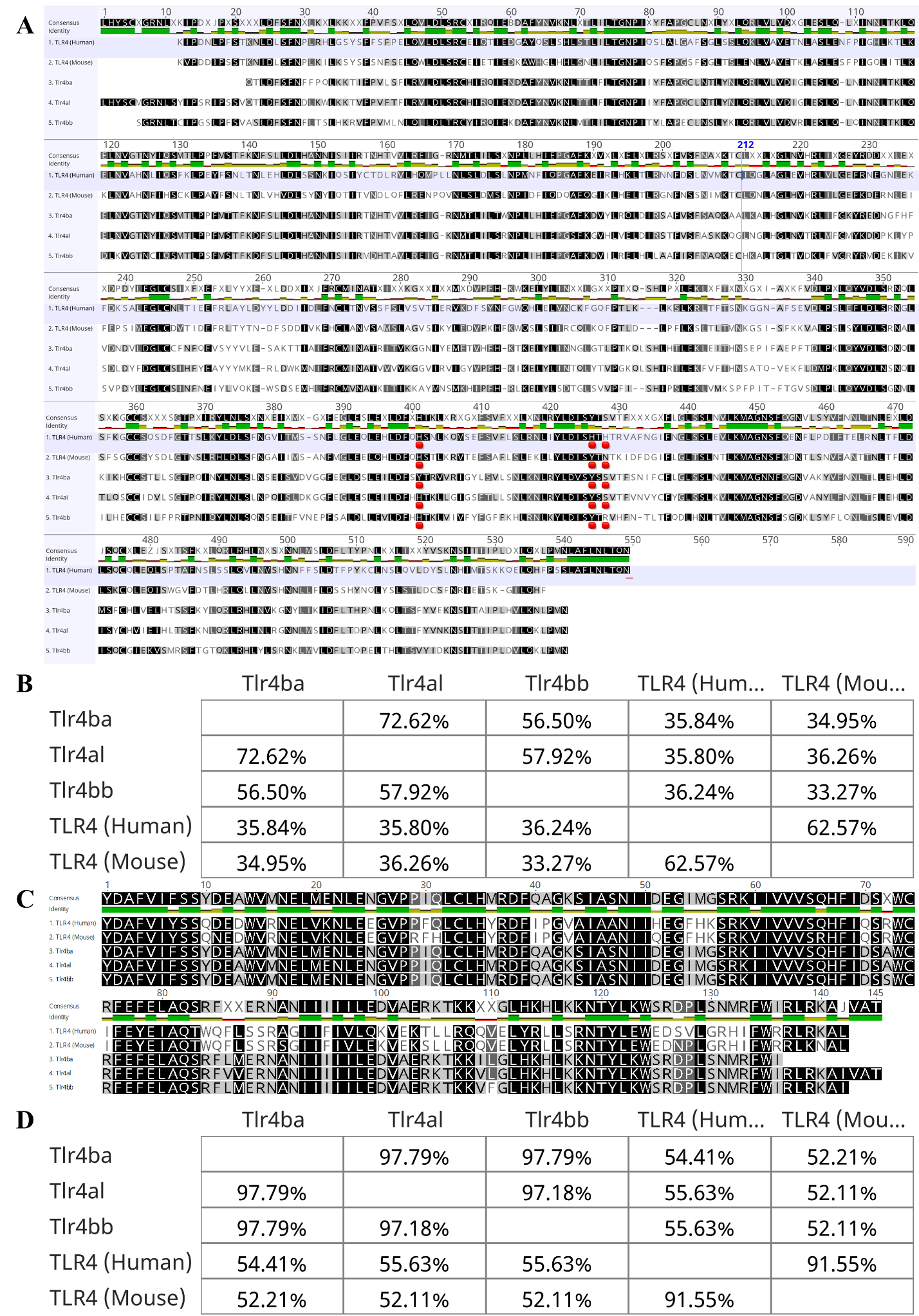
**

**Supplementary Figure 2. Multiple sequence alignment and percent identity matrix for TLR4 extracellular LRR domain and intracellular TIR domain between human, mouse, and zebrafish.** The comparison between extracellular LRR domain (A, B) sequences shows the homology and conservation of residues is ~20% greater within the intracellular TIR domain (C, D) compared with the extracellular LRR domain. Similar residues (100%) are highlighted in black, partially similar residues are highlighted in dark grey (80%-100%) and light grey (60%-80%). The coloured histogram shows the mean pairwise percent identity for each column of the alignment, where green represents 100%, yellow represents >30%, and red represents <30%. The known human nickel binding residues are underlined in red within A. The nickel binding residues 431 and 456 show 100% similarity between humans, mice, and zebrafish, as well as partial identity (>30%) and consist of either histidine or tyrosine residues. The last known nickel binding residue (458) shows low similarity and identity between all sequence and is either a histidine, asparagine, serine, or arginine residue. The tables (B, D) below the alignments (A, C) show the percent sequence identity for the entire sequence alignment between human TLR4, mouse TLR4, zebrafish Tlr4ba, Tlr4al, and Tlr4bb. Alignments, similarity, and the percent identity matrix were made and calculated using Geneious Prime.


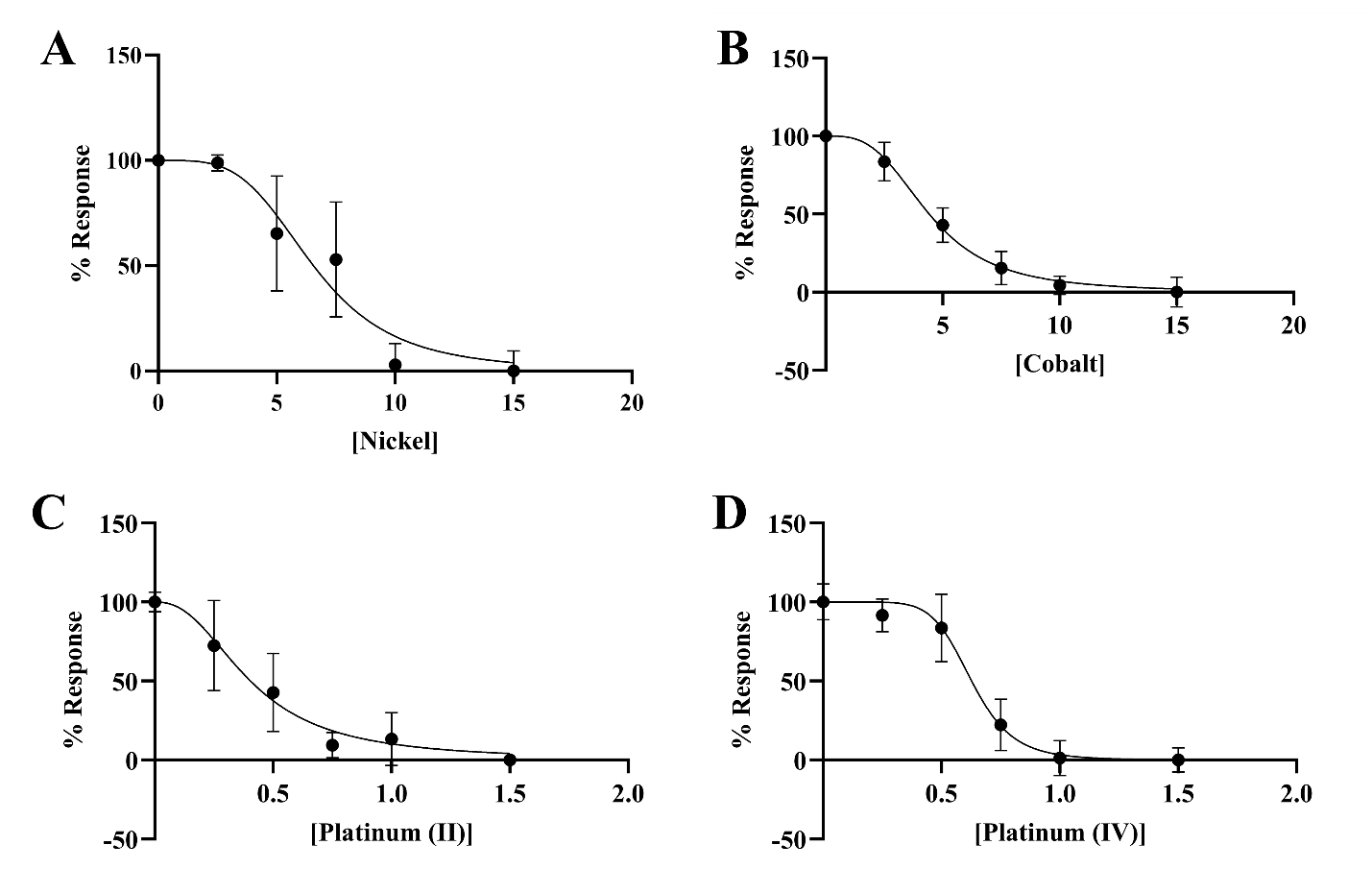


**Supplementary Figure 3. Normalized linear regression plots of nickel, cobalt, platinum II, and platinum IV dose response curves.** Neuromast viability responses to varying concentrations of NiCl_2­_ (A), CoCl_2_ (B), PtCl_2_ (C), and PtCl_4_ (D) were averaged and normalized where 0% and 100% of a response was defined as the smallest and largest mean in each data set respectively. EC_50_ values were then calculated using a non-linear regression with variable slope in GraphPad Prism 10.

**
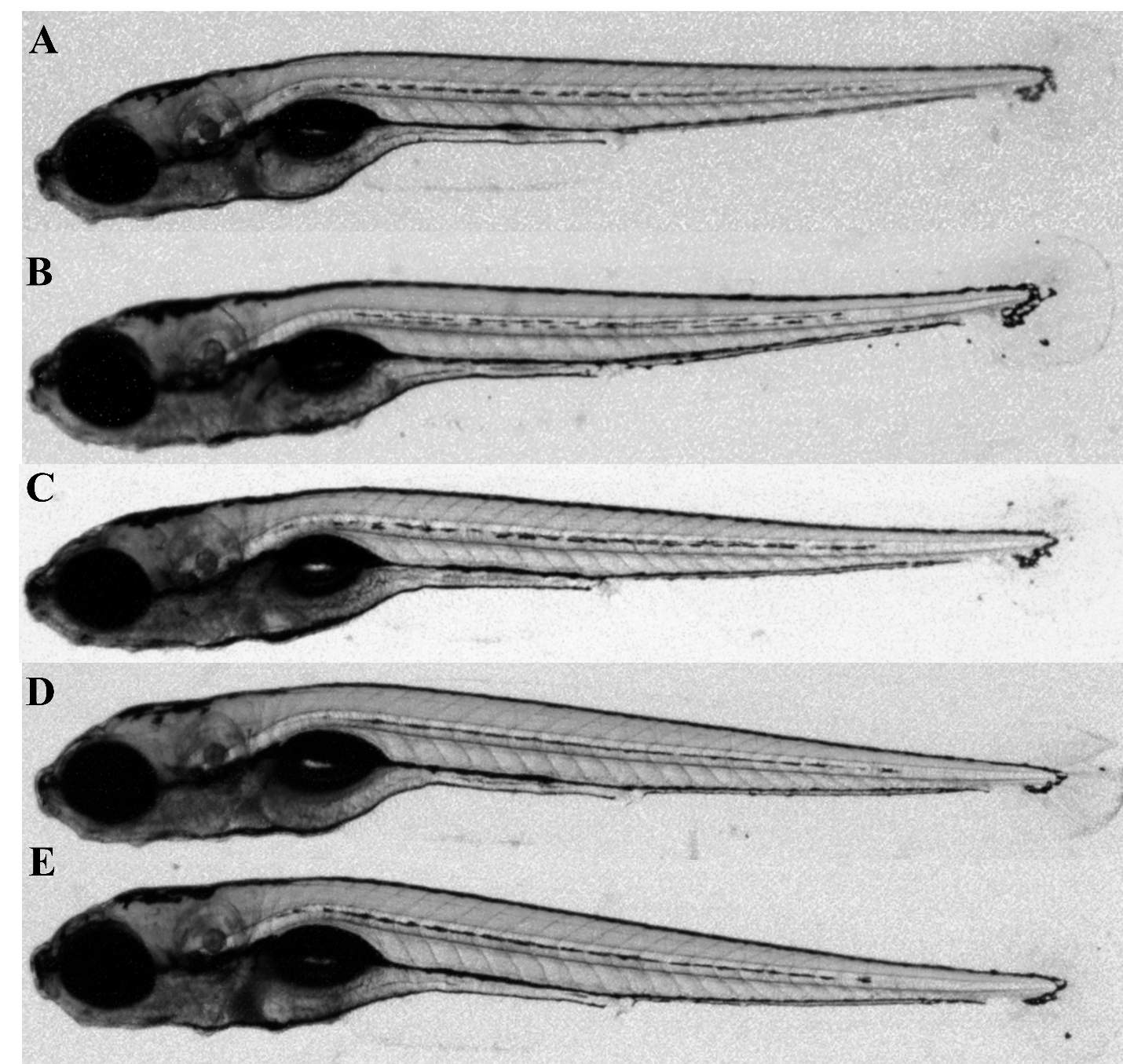
**

**Supplementary Figure 4. Tlr4 crispant fish treated with nickel (II) and platinum (IV).** A-C) 6-7dpf mock injected and Tlr4 crispant larva treated with either PtCl_4_ or NiCl_2_ show no apparent morphological change in comparison to an uninjected untreated larva. A) Uninjected, untreated larva. B) Mock injected larva treated with 7.5µM PtCl_4_. C) Tlr4 crispant treated with 7.5µM PtCl_4_. D) Mock injected larva treated with 10µM NiCl_2_. E) Tlr4 crispant larva treated with 10µM NiCl_2_.

**
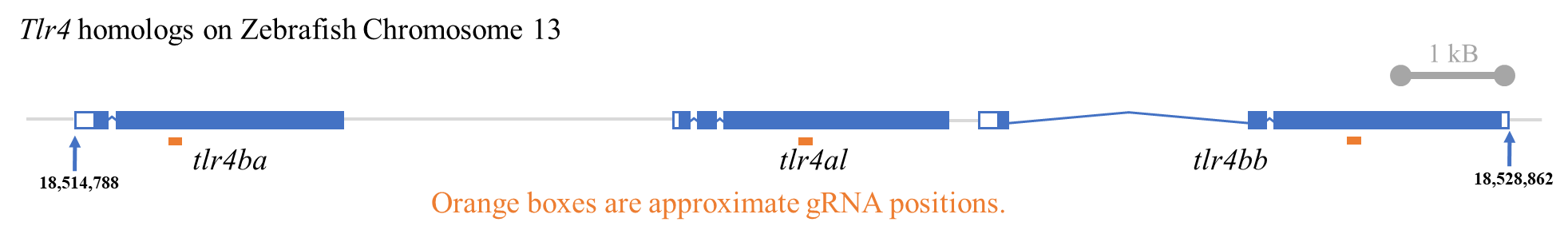
**

**Supplementary Figure 5.** **Illustration representing the three *TLR4* homologs on zebrafish chromosome 13 and their approximate gRNA positions.** The three zebrafish *tlr4* homologs are found in succession to one another. Arrows point to the initial base pair position on the chromosome. White boxes represent untranslated regions, blue boxes represent exons, while blue lines denote introns. Grey lines represent the intergenic region connecting the homologs.

**
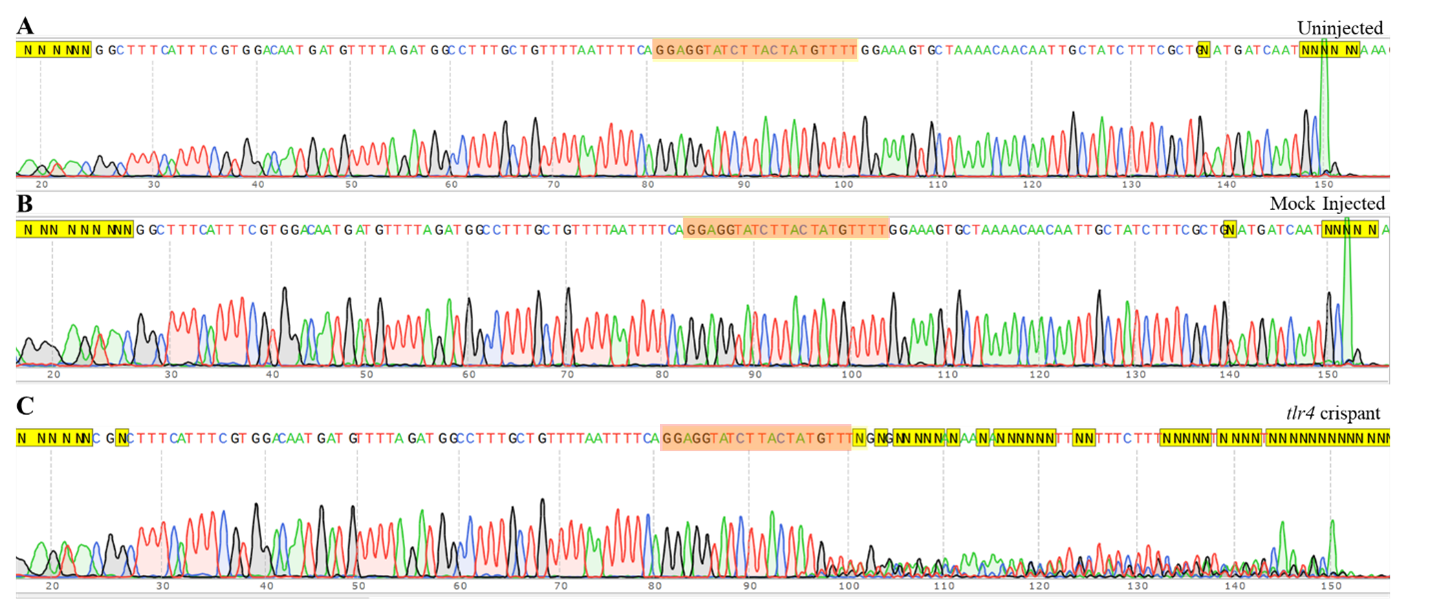
**

**Supplementary Figure 6. Sanger Sequencing of tlr4ba crispant DNA.** The *tlr4ba* gene was amplified from the genomic DNA of individual larvae via PCR using the tlr4ba forward primer. Uninjected larva (A) and mock injected larva (B) DNA shows an unmutated *tlr4ba* sequence, where consistent DNA in all cells produces a coherent chromatogram throughout. C) Chromatograms from *tlr4* cripants show consensus sequence chromatograms where the DNA is homozygous and consistent throughout the individual (left side), but the chromatogram becomes discrepant from wild type sequence near the gRNA binding site (highlighted in red) because a mixture of various mutant DNA sequences now exists among the larva’s cells. Larvae were injected at the single-cell stage with either a gRNA sequence targeting the zebrafish tlr4ba homolog or Cas9 protein alone (mock).

**
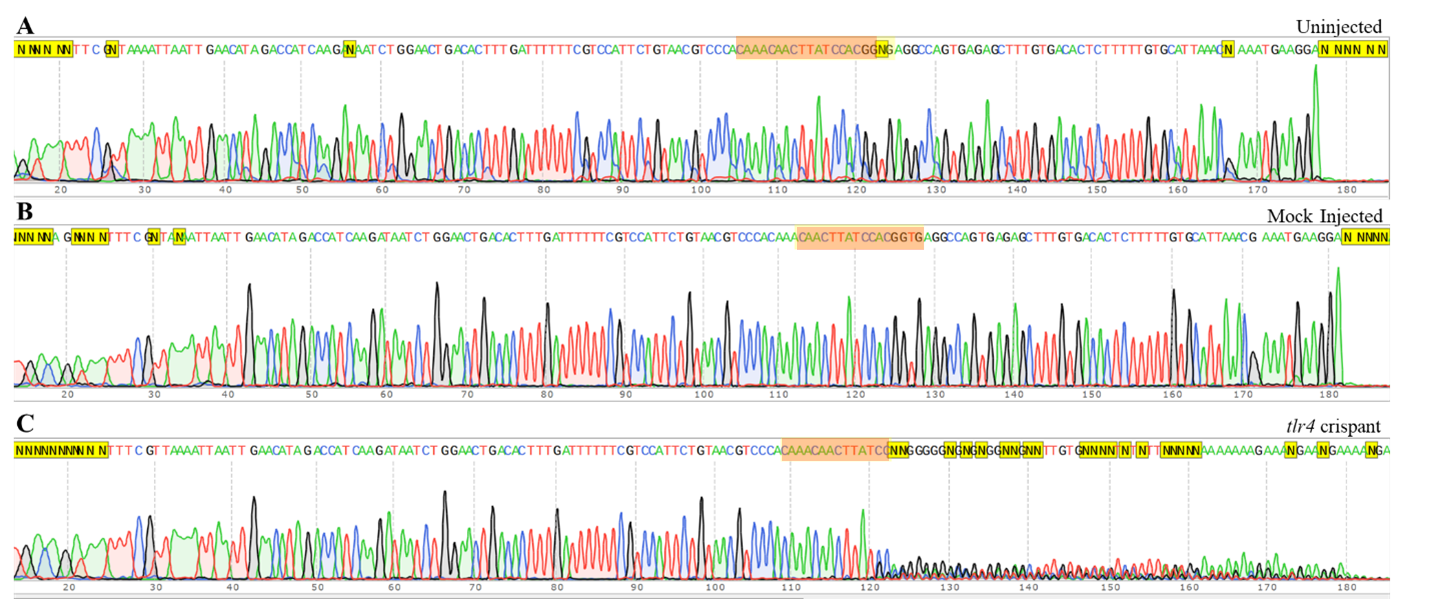
**

**Supplementary Figure 7. Sanger Sequencing of tlr4bb crispant DNA.** The *tlr4bb* gene was amplified from the genomic DNA of individual larvae via PCR using the *tlr4bb* reverse primer. Uninjected larva (A) and mock injected larva (B) DNA shows an unmutated *tlr4bb* sequence, where consistent DNA in all cells produces a coherent chromatogram throughout. C) Chromatograms from *tlr4* crispants show consensus sequence chromatograms where the DNA is homozygous and consistent throughout the individual (left side), but the chromatogram becomes becomes discrepant from wild type sequence near the gRNA binding site (highlighted in red) because a mixture of various mutant DNA sequences now exists among the larva’s cells. Larvae were injected at the single-cell stage with either a gRNA sequence targeting the zebrafish tlr4bb homolog or Cas9 protein alone (mock).

**
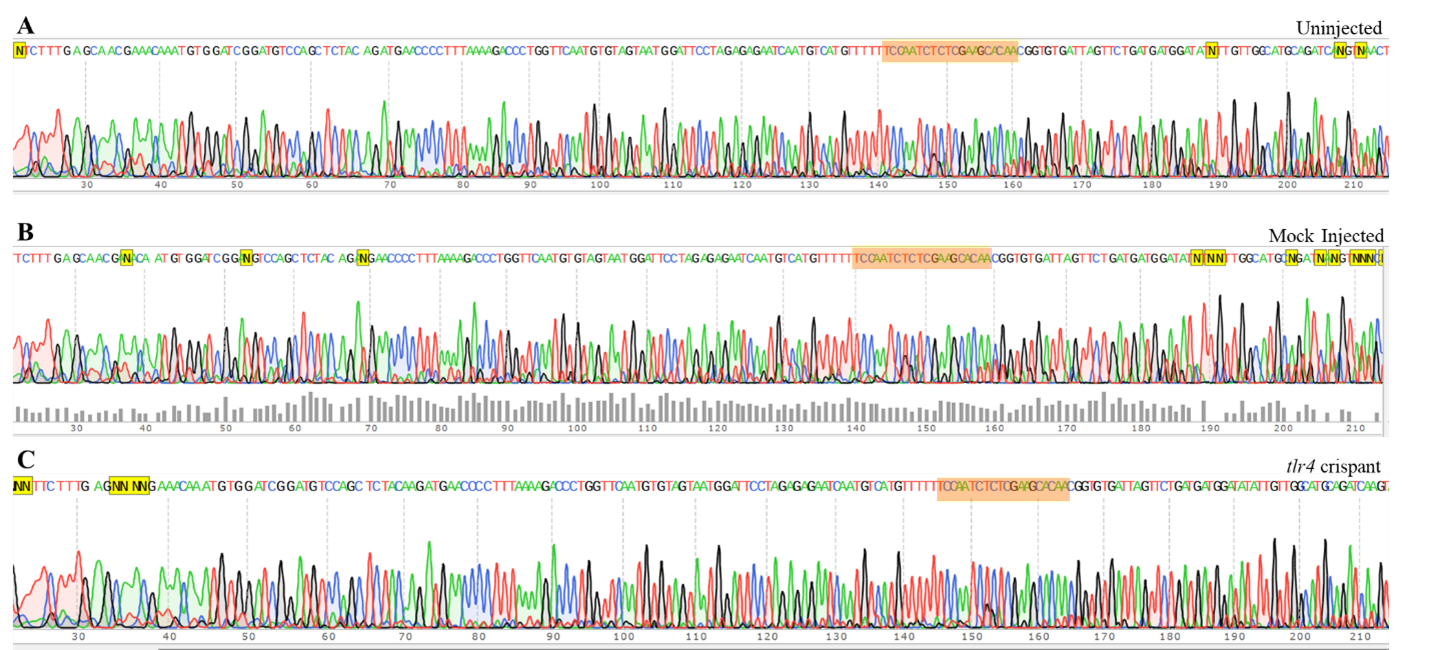
**

**Supplementary Figure 8. Sanger Sequencing of tlr4al crispant DNA.** The *tlr4al* gene was amplified from the genomic DNA of individual larvae via PCR using the *tlr4al* reverse primer. Uninjected larva (A) and mock injected larva (B) DNA in A & B shows an unmutated *tlr4al* sequence, where consistent DNA in all larval cells produces a coherent chromatogram throughout. C) Chromatograms from *tlr4* cripants show consensus sequence chromatograms where the DNA is homozygous and consistent throughout the individual even following the gRNA target sequence (highlighted in red).

**
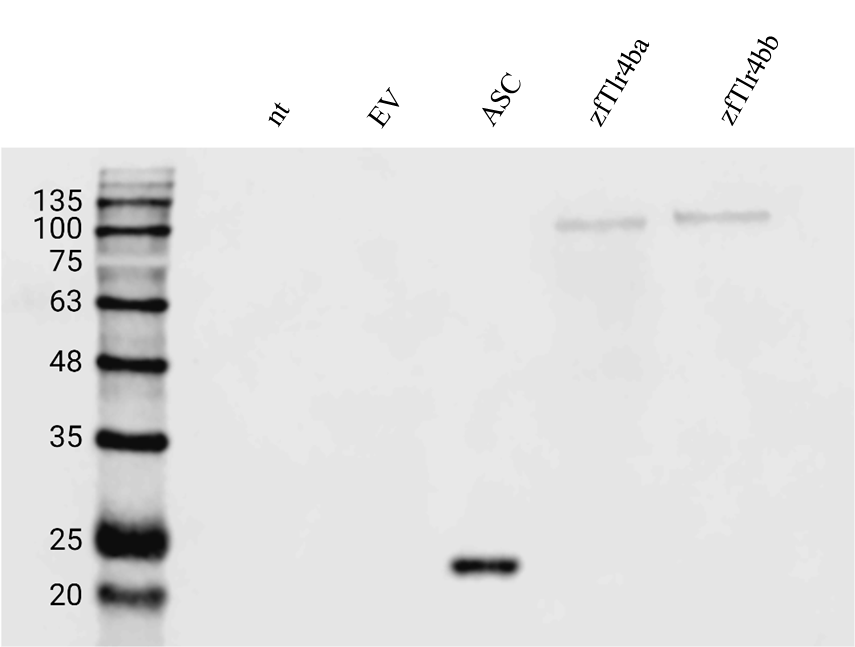
**

**Supplementary Figure 9.** **Zebrafish Tlr4ba and Tlr4bb are stably produced in HEK293T cells.** Zebrafish *tlr4ba* and *tlr4bb* were transiently expressed in HEK293T cells from a constitutive promoter. A signal of the expected size was observed in cells transiently expressing V5/His_6_-epitope tagged zebrafish Tlr4 proteins (zfTlr4ba, zfTlr4bb). Lysate from cells transiently expressing V5-tagged ASC was used as a positive control, while lysates from non-transfected (nt) or empty vector (EV)-transfected HEK293T cells were used as negative controls.

**Supplementary Table 1. CRISPR-cas9 gRNA sequences and PCR primer sequences.**

| **Gene** | **gRNA sequence** | **PCR primer sequences** |
| --- | --- | --- |
| ***tlr4ba*** | 5’-GGAGGUAUCUUACUAUGUUUGUUUUAGAGCUAU-3’ | F: 5’-CCTGCATGGTCTTAATGTCAA-3’ |
|  |  | R: 5’-GCATTGATCATACAGCGAAAGA-3’ |
| ***tlr4al*** | 5’-UCCAAUCUCUCGAAGCACAAGUUUUAGAGCUAUGCU-3’ | F: 5’-GACTCTTCCTCCATTCATGAGC-3’ |
|  |  | R: 5’-CAAACATCAGCCTTGTGACATT-3’ |
| ***tlr4bb*** | 5’-CAAACAACUUAUCCACGGUGGUUUUAGAGCUAU-3’ | F: CCTTCATTTCGTTTAATGCACA-3’ |
|  |  | R: AACGAAACAAGTGCATTTCTGA- 3’ |
